# Psychosomatic profile in patients with systemic sclerosis: results from an observational study

**DOI:** 10.3389/fmed.2025.1631450

**Published:** 2025-07-08

**Authors:** Sara Romanazzo, Francesco Sera, Margherita Cappelli, Francesca Nacci, Serena Guiducci, Fiammetta Cosci

**Affiliations:** ^1^Department of Health Sciences, University of Florence, Florence, Italy; ^2^Department of Statistics, Computer Science and Applications G. Parenti, University of Florence, Florence, Italy; ^3^Rheumatology Unit, Department of Experimental and Clinical Medicine, University of Florence, Florence, Italy; ^4^Department of Psychiatry and Neuropsychology, Maastricht University, Maastricht, Netherlands

**Keywords:** psychological distress, well-being, resilience, systemic sclerosis, DCPR

## Abstract

**Objectives:**

The aim of the present study was to test whether the occurrence of psychosomatic syndromes in patients with systemic sclerosis (SSc) may influence psychopathological distress and well-being.

**Methods:**

A total of 276 outpatients with SSc were consecutively enrolled. Mental disorders were assessed using the Mini-International Neuropsychiatric Interview (MINI), while psychosomatic syndromes were assessed using the Semi-Structured Interview for Diagnostic Criteria for Psychosomatic Research-Revised (DCPR-R-SSI). Psychological distress and well-being were assessed using the Mental Pain Questionnaire (MPQ), the Symptom Questionnaire (SQ), the Psychological Well-Being (PWB) scales, the 5-item World Health Organization Well-Being Index (WHO-5), the Euthymia Scale (ES), and the Pictorial Representation of Illness and Self Measure (PRISM). Latent Class Analysis (LCA) was performed on the 14 items of the DCPR-R-SSI. The LCA solution identified two distinct latent patient groups with distinct clinical profiles: LC1, comprising 255 patients (92.4%), and LC2, comprising 21 patients (7.6%).

**Results:**

DCPR-R allostatic overload, demoralization, irritable mood, type a behavior, and alexithymia primarily discriminated between the two distinct latent groups of patients. The probabilities of observing these syndromes were higher among the patients belonging to the LC2 group. Depression was found to be associated with belonging to the LC2 group, as well as with higher scores on the MPQ and the SQ scales for depression, anxiety, anger-hostility, and somatization (*p* < 0.05). In addition, lower scores were observed on the PWB scales for environmental mastery, positive relationships with others, purpose in life, and self-acceptance, as well as on the WHO-5, ES, and PRISM measures of feeling at peace (*p* < 0.05).

**Conclusion:**

Psychosomatic syndromes may help define distinct clusters among patients with SSc, reflecting specific clinical profiles that should be considered during patient assessment and when proposing tailored interventions.

## 1 Introduction

Systemic sclerosis (SSc) is a rare autoimmune connective tissue disease characterized by fibrosis of the skin and internal organs. Fibrosis leads to changes in physical appearance and functional disability, which can result in significant limitations in social functioning and psychological distress (1). A substantial body of literature suggests that patients with SSc are at increased risk of developing mental disorders, particularly depression and anxiety (2–4), and tend to have poor psychological well-being (4). On the contrary, no evidence is available on the occurrence of psychosomatic syndromes in SSc—syndromes that are not included in the standard nosography (i.e., the Diagnostic and Statistical Manual of Mental Disorders—DSM) but are clinically relevant according to the bio-psycho-social model (5).

Psychosomatic syndromes have been extensively assessed using the Diagnostic Criteria for Psychosomatic Research (DCPR) (6) and its revised version (DCPR-R) (7). The DCPR is a clinimetric tool complementary to the DSM, designed to comprehensively investigate (and make the corresponding diagnosis of) syndromes that are at the intersection of biological, psychological, and social factors. A semi-structured interview for the DCPR-R was also developed (7).

The DCPR have been applied in various medical settings to assess the prevalence of psychosomatic syndromes in medically ill patients. Patients with conditions such as fibromyalgia, migraine, irritable bowel syndrome, and coronary heart disease showed DCPR-R syndrome prevalences of 78, 55, 89.7, and 51.3%, respectively (8–11). The most common diagnoses included allostatic overload, alexithymia, type A behavior, and persistent somatization (8–13). In addition, medically ill patients with fibromyalgia, migraine, irritable bowel syndrome, coronary heart disease, or type 2 diabetes who had at least one DCPR psychosomatic syndrome were found to have higher psychological distress, poorer well-being, reduced health-related quality of life, and impaired psychosocial functioning compared to those without a psychosomatic syndrome (9–13). Similar results were observed in older individuals from the general population (14). In medical settings, DCPR diagnoses (i.e., irritability, demoralization, persistent somatization, functional somatic symptoms secondary to a mental disorder, conversion symptoms, anniversary reactions, and illness behavior) helped define patient clusters with specific clinical features that warrant clinical attention due to psychological distress and illness behavior (15). Interestingly, in a study on patients with obesity, the level of psychological well-being decreased with the increasing number of psychosomatic syndromes diagnosed (16), thereby suggesting that a higher number of psychosomatic syndromes is associated with greater psychological distress and reduced well-being.

The aim of the present study was to determine the prevalence of DCPR-R psychosomatic syndromes in patients with SSc and to assess whether variations in the number of psychosomatic syndromes within this specific clinical population are associated with differing levels of psychopathological distress and psychological well-being.

## 2 Materials and methods

### 2.1 Participants and procedure

A total of 276 outpatients with SSc were consecutively enrolled at the Scleroderma Unit of the Rheumatology Department, University Hospital Careggi (Florence, Italy), from June 2020 to September 2022. The inclusion criteria were as follows: (a) age of at least 18 years; (b) diagnosis of systemic sclerosis according to the 2013 American College of Rheumatology (ACR) and the European League Against Rheumatism (EULAR) classification (17); and (c) native Italian speaker. The exclusion criteria were as follows: (a) changes in pharmacological therapy within the past 3 months (as documented in the clinical records) and (b) cognitive deficits or other impairments affecting patients’ ability to follow the study procedures.

The participants were evaluated by trained clinical psychologists using the Mini-International Neuropsychiatric Interview (MINI) (18) and the Semi-Structured Interview for Diagnostic Criteria for Psychosomatic Research-Revised (DCPR-R-SSI) (7). The following self-report tools were administered: the Health Assessment Questionnaire Disability Index (HAQ-DI) (19), the Mental Pain Questionnaire (MPQ) (20), the Symptom Questionnaire (SQ) (21), the Psychological Well-Being (PWB) scales (22), the 5-item World Health Organization Well-Being Index (WHO-5) (23, 24), the Euthymia Scale (ES) (25), and the Pictorial Representation of Illness and Self Measure (PRISM) (26). Sociodemographic and clinical information was collected using *ad hoc* questions that had been previously developed and used in past studies (27).

Participation was voluntary and not compensated. All participants provided and signed written informed consent, including a privacy protection disclaimer. The study protocol was approved by the Regional Ethical Committee for Clinical Experimentation of the Tuscan Region in Florence, Italy (protocol code: WBTinSSC, date: 25.02.2020).

### 2.2 Instruments

The MINI is a brief, structured diagnostic interview designed to assess 17 mental disorders according to the DSM and the International Classification of Diseases (18). It includes 120 items with a “yes” or “no” closed-response format to determine whether the diagnostic criteria for a mental disorder are met. The MINI is an accurate tool with high reliability and validity (28).

The DCPR-R-SSI (7) is a semi-structured interview based on the Diagnostic Criteria for Psychosomatic Research-Revised (7), developed to diagnose 14 psychosomatic syndromes: allostatic overload, health anxiety, disease phobia, hypochondriasis, thanatophobia, illness denial, persistent somatization, conversion symptoms, anniversary reaction, somatic symptoms secondary to a psychiatric disorder, demoralization, irritable mood, type A behavior, and alexithymia. The interview focuses on the previous 6–12 months and includes 79 “yes/no” items. The DCPR-R-SSI has demonstrated good criterion-related validity (9), incremental validity (14), inter-rater reliability, and concurrent validity (29).

The HAQ-DI is a widely used self-assessment tool for measuring functional disability (19). It includes 20 items scored on a 4-point Likert scale assessing specific activities of daily living, grouped into eight functional categories (i.e., dressing, rising, eating, walking, hygiene, reach, grip, and usual activities). Each item is scored from 0 (“without difficulty”) to 3 (“unable to do”). The scores of these domains are averaged, resulting in a total disability index score of 0–3. Increasing scores indicate worse functioning. A HAQ-DI score of 0 indicates no functional disability, while a score of 3 indicates severe functional disability (30). The HAQ-DI has shown good validity and reliability (31).

The MPQ is a 10-item self-report questionnaire assessing mental pain over the previous week (20). The items are rated using a dichotomous response format (i.e., yes/true = 1; no/false = 0), with a total score ranging from 0 to 10. Higher total scores indicate more severe mental pain. The MPQ has shown good clinimetric properties (20, 32).

The SQ is a 92-item self-report questionnaire assessing psychological distress over the previous week (21). It has four scales measuring depression, anxiety, anger-hostility, and somatization. Each scale has two subscales—one related to symptoms and one related to well-being—resulting in a total of eight subscales. The brief and simple items are formulated using a dichotomous response format (i.e., yes/true = 1; no/false = 0). The scales and subscales can be scored separately, with higher scores indicating higher distress. The SQ has demonstrated adequate discriminant validity, concurrent validity, and predictive validity (33).

The PWB scales are an 84-item self-administered questionnaire assessing six domains of psychological well-being: autonomy, environmental mastery, personal growth, positive relationships with others, purpose in life, and self-acceptance (22). The items are rated on a 6-point Likert scale ranging from 1 (“strongly disagree”) to 6 (“strongly agree”). The total score ranges from 14 to 84; higher scores indicate higher psychological well-being. The PWB scales have shown good clinical sensitivity (34) and reliability (22).

The WHO-5 is a 5-item self-administered global rating scale measuring subjective well-being over the previous 14 days (23). Each item is scored on a 6-point Likert scale, with responses ranging from 5 (“all of the time”) to 0 (“none of the time”). The raw score ranges from 0 (absence of well-being) to 25 (maximal well-being) and is multiplied by 4 to yield a percentage score (i.e., from 0 = absent to 100 = maximal) (35). The WHO-5 has demonstrated good construct validity, predictive validity, sensitivity, and specificity (24, 35).

The ES is a 10-item self-rating scale assessing euthymia (25). Five items were derived from the WHO-5 (23), while the other five were created with the purpose of measuring psychological flexibility, consistency, and resilience. Each item is scored on a 6-point Likert scale ranging from 1 (“strongly disagree”) to 6 (“strongly agree”) (25). Total scores range between10 and 60, with higher scores indicating higher levels of euthymia. The ES has demonstrated construct validity and clinimetric sensitivity (25).

**Figure 1:**
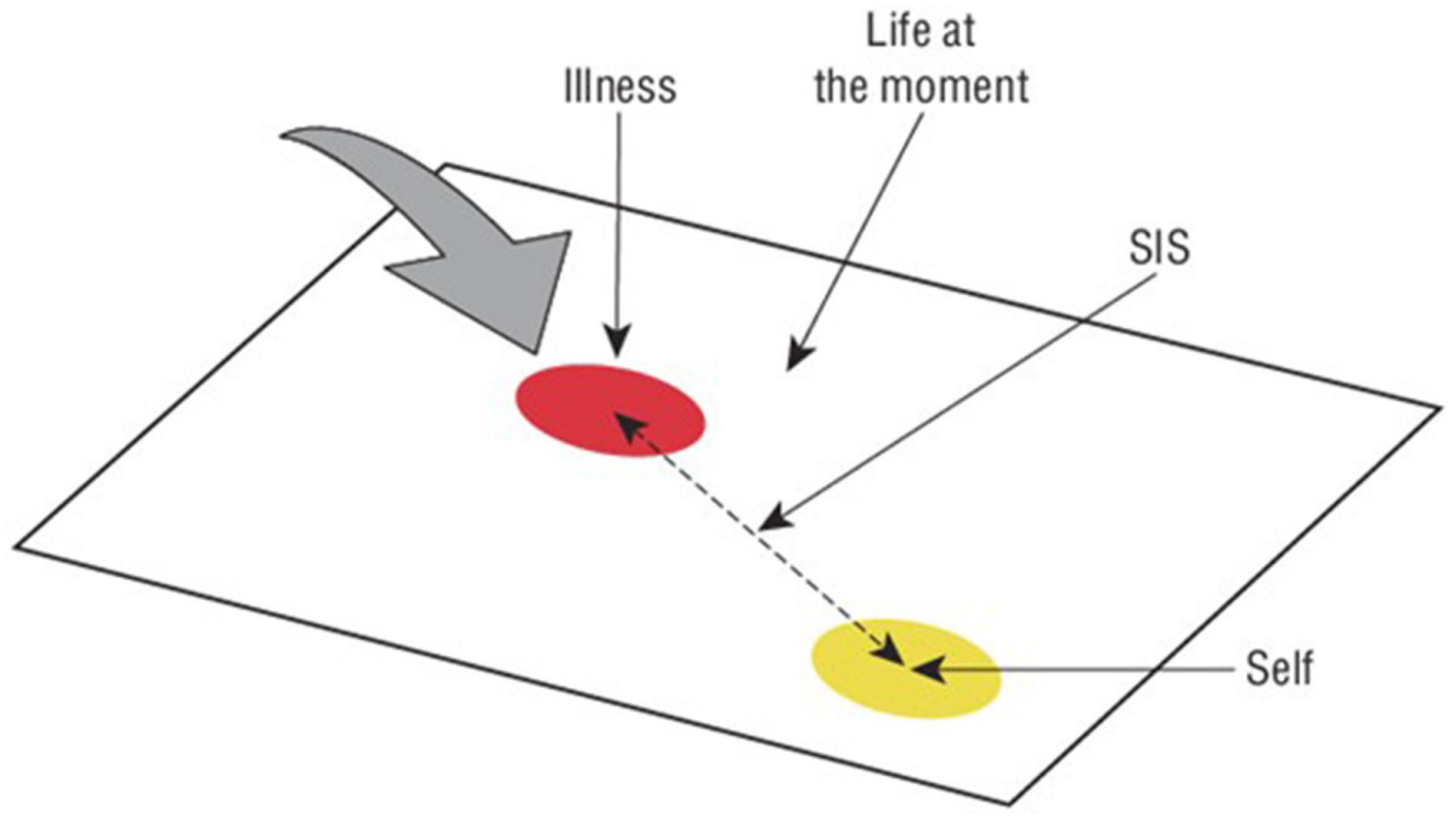
Pictorial representation of illness and self measure (PRISM).

The PRISM is a simple visual tool used to assess the burden or the invasiveness of illness in a patient’s life (26). Participants are shown a white A4-size sheet of paper with a yellow disk (diameter = 7 cm) fixed to the bottom right-hand corner. They are asked to imagine that the board represents their current life and the yellow disk represents themselves. Participants then receive a red disk (diameter = 5 cm) representing their illness and are asked: “Where would you place the illness-disk in your life right now?.” The primary PRISM outcome measure is the self-illness separation (SIS), that is the distance (in centimeters) between the centers of the self-disk and the illness-disk (SIS range = 0–27 cm) (see Figure 1); smaller values mean greater distress (36). Additional colored disks may be used to represent other aspects of the patient’s life (i.e., family, work, hobbies, and friends) (37). In the present study, additional colored disks were used to assess physical pain, feeling at peace, and leisure activities. The PRISM has shown good content validity and test–retest reliability (37).

### 2.3 Statistical analysis

To identify groups with similar psychosomatic profiles, a Latent Class Analysis (LCA) (38) was performed on the 14 items of the DCPR-R-SSI. LCA belongs to a family of statistical procedures used to uncover hidden groups. It is specifically designed for categorical variables, and its main assumption is that the observed (or “manifest”) variables are statistically independent, conditional on the latent classes. In brief, the latent class “explains” the associations among the manifest categorical variables.

The joint distribution of the observed responses $Y_{i}$ is modeled as a weighted sum of the conditional probabilities of the responses, with the weights given by the population proportions in each class.


$$P(Y_{i}|p)=\sum_{r=1}^{R}p_{r}\prod_{j=1}^{J}\prod_{k=1}^{K_{j}}{\left(\pi_{\mathrm{jrk}}\right)}^{Y_{\mathrm{ijk}}}$$

Where.

- $p_{r}$ is the proportion of the population belonging to class 𝑟;
- $\pi_{\mathrm{jrk}}$ is the conditional probability, for class 𝑟, of observing the 𝑘-th response on the 𝑗-th variable;
- $Y_{\mathrm{ijk}}$ is the indicator variable that assumes the value 1 if individual 𝑖 has given answer 𝑘 to variable 𝑗; otherwise it assumes 0.

Note that in this application, J=14 (the number of the DCPR-R-SSI instrument items) and $K_{j}=2$, as each item represents the presence/absence of the 14 psychosomatic syndromes.

Once the parameters $p_{r}$ and $\pi_{\mathrm{jrk}}$ have been estimated for each individual, the posteriori probability of belonging to latent class *r*, given its observed pattern of responses $Y_{i}$, can be calculated using the following formula based on Bayes’s Theorem:


$$\hat{P}(r|Y_{i})=\frac{{\hat{p}}_{r}f(Y_{i};{\hat{\pi }}_{r})}{\sum_{q=1}^{R}{\hat{p}}_{q}f(Y_{i};{\hat{\pi }}_{q})}$$

To identify the number of hidden groups, we fitted a series of LCA models with 1 to 10 latent classes and compared them using the Bayesian information criterion (BIC) and Akaike information criterion (AIC) (39). After selecting the k-class model with the lowest relative model fit values, each participant was assigned to a class based on their highest posterior probability of belonging to each of the k-classes. We then carried out an analysis to interpret the identified classes. The prevalence of the latent classes was compared by age, sex, educational level, marital status, and diagnosis of major depressive disorder (MDD). A chi-squared test was used to evaluate the associations between these categorical variables and latent classes. Means and standard deviations of the HAQ-DI, MPQ, SQ, PWB, WHO-5, ES, and PRISM scores were calculated according to the latent classes, and mean differences were tested using the *t*-test (with two latent classes).

## 3 Results

**Figure 2:**
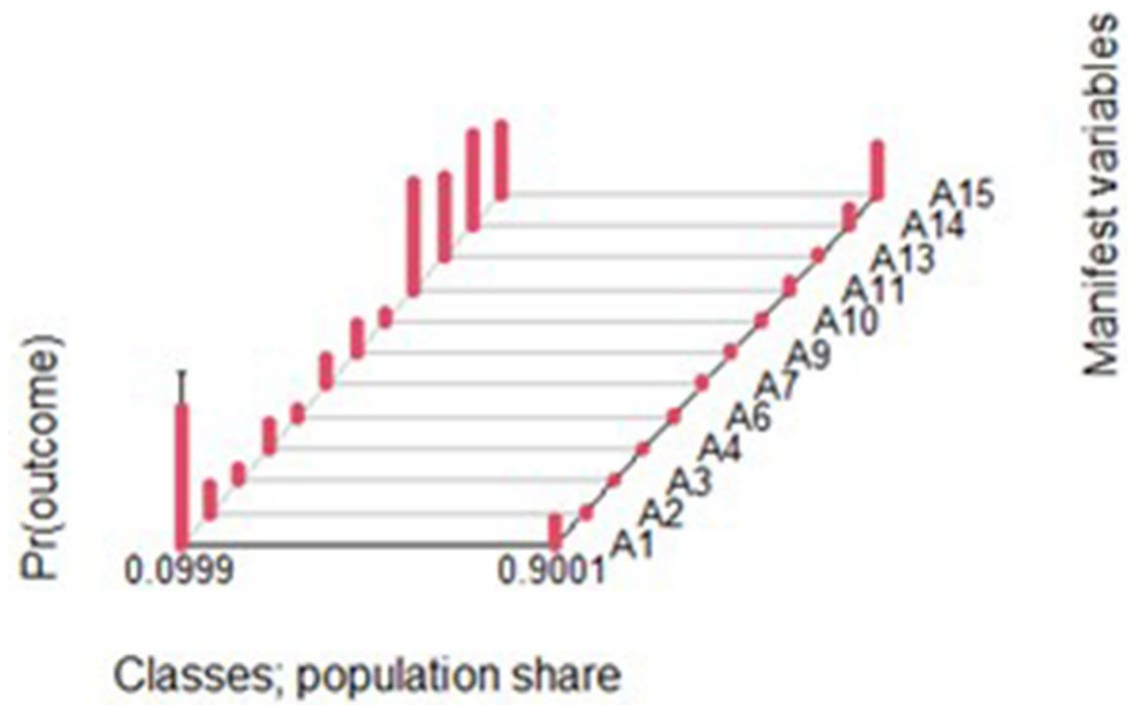
Probability of the DCPR diagnoses occurrence: 2 latent groups identified via the LCA notes: Each psychosomatic syndrome is labelled with an A letter as manifest variable. Pr 0.0999 refers to LC2 group. Pr 0.9001 refers to LC1 group.

A total of 276 patients diagnosed with SSc were analyzed. Using the BIC, the LCA solution identified two distinct latent patient groups with different clinical profiles: LC1, which included 255 patients (92.4%), and LC2, which included 21 patients (7.6%). The DCPR-R-SSI items that most strongly discriminated between the two groups were as follows: allostatic overload (named A1 in Figure 2), demoralization (A11), irritable mood (A13), type A behavior (A14), and alexithymia (A15). The probabilities of observing these items were higher in the patients belonging to LC2 (see Figure 2).

Age was associated with belonging to LC2, with a higher prevalence among the participants younger than 55 years (*p* = 0.006) (see Table 1). Major depressive disorder (MDD)—including major depressive single and/or recurrent episodes diagnosed via the MINI—was also associated with belonging to LC2 (*p* = 0.015) (see Table 1).

**Table 1:** Differences in demographic and clinical characteristics between the two latent classes.

	LC1 (*n* = 255)	LC2 (*n* = 21)	*p*
Age, *n* (%)			
<55	73 (84.9)	13 (15.1)	0.006
55–65	73 (94.8)	4 (5.2)	
≥65	109 (96.5)	4 (3.5)	
Sex, *n* (%)			
Female	229 (93.1)	17 (6.9)	0.210
Male	26 (86.7)	4 (13.3)	
Education, *n* (%)			
Primary/secondary school	91 (91.9)	8 (8.1)	0.937
High school	107 (92.2)	9 (7.8)	
University degree	57 (93.4)	4 (6.6)	
Marital status, *n* (%)			
Married	171 (94.0)	11 (6.0)	0.173
Not married	84 (89.4)	10 (10.6)	
MINI MDE/DDM			
Yes	51 (85.0)	9 (15.0)	0.015
No	204 (94.4)	12 (5.6)	

Total numbers are presented, with percentages in brackets. MINI, Mini-International Neuropsychiatric Interview; MDE, major depressive episode; MDD, major depressive disorder.

Despite similar levels of systemic sclerosis severity (HAQ-DI; *p* = 0.348), the participants in LC2 showed higher scores than those in LC1 on the MPQ and the SQ scales for depression, anxiety, anger-hostility, and somatization (*p* < 0.05) and lower scores on the PWB scales for environmental mastery, positive relationships with others, purpose in life, and self-acceptance, the WHO-5, the ES, and the PRISM measure of feeling at peace (*p* < 0.05) (see Table 2).

**Table 2:** Differences in psychological distress and well-being levels between the two latent classes.

	LC1		LC2		*p*
	**Mean**	**SD**	**Mean**	**SD**	
HAQ-DI functional disability	0.53	0.62	0.69	0.72	0.348
MPQ total score	1.4	2.0	4.2	2.5	<0.001
SQ depression	4.5	3.9	10.4	7.2	0.001
SQ anxiety	4.7	4.1	11.1	7.6	0.001
SQ anger-hostility	9.2	8.0	21.5	14.8	0.001
SQ somatization	13.9	12.0	32.6	22.4	0.001
PWB autonomy	64.3	10.1	59.2	11.4	0.058
PWB environmental mastery	61.1	10.5	55.1	11.8	0.034
PWB personal growth	60.9	9.6	58.1	11.6	0.302
PWB positive relationships	65.1	10.8	54.4	13.2	0.002
PWB purposes in life	61.6	10.0	54.3	14.6	0.035
PWB self-acceptance	62.1	11.2	49.2	14.1	<0.001
WHO-5 total score	58.6	21.5	38.3	20.1	<0.001
ES total score	43.4	10.2	35.1	10.8	0.002
PRISM illness	9.9	6.1	9.0	5.3	0.440
PRISM physical pain	12.0	6.7	10.5	6.9	0.343
PRISM feeling at peace	8.3	5.3	13.9	7.0	0.014
PRISM leisure activities	9.2	5.8	11.6	8.1	0.097

HAQ-DI, Health Assessment Questionnaire Disability Index; MPQ, Mental Pain Questionnaire; SQ, Symptom Questionnaire; PWB, Psychological Well-Being Scales; WHO-5, 5-item World Health Organization Well-Being Index; ES, Euthymia Scale; PRISM, Pictorial Representation of Illness and Self Measure.

## 4 Discussion

The study showed the existence of two different groups of patients with SSc with similar levels of functional disability but different psychosomatic profiles, characterized by a higher occurrence of DCPR allostatic overload, demoralization, irritable mood, type A behavior, and alexithymia. The mentioned psychosomatic syndromes discriminated between the two groups as they had a higher probability of being detected in LC2 than in LC1. Major depressive disorder was also associated with the LC2 group at a higher probability than with LC1. The LC2 patients also showed higher psychological distress, characterized by mental pain, anxiety, anger-hostility, somatization, and lower well-being. Well-being was assessed in terms of environmental mastery, positive relationships with others, purpose in life, and self-acceptance, as well as WHO-5 subjective well-being, euthymia, and the PRISM measure of feeling at peace.

This is the first study investigating the occurrence of psychosomatic syndromes in systemic sclerosis; therefore, the results cannot be compared with findings from previous studies in the same clinical population. Referring to different clinical samples used as proxies, similar results were found in heart-transplanted patients—who exhibited DCPR demoralization, type A behavior, irritable mood, and alexithymia (40); in patients with migraine—characterized by high rates of allostatic overload, type A behavior, irritable mood, and alexithymia (9); and in patients with human papillomavirus infection (41), irritable bowel syndrome (42), fibromyalgia, coronary heart disease, and type 2 diabetes (43).

A significant number of psychosomatic syndromes was found to affect the LC2 group, characterized by allostatic overload, demoralization, irritable mood, type A behavior, and alexithymia. In addition to such syndromes, clinical features such as a diagnosis of major depressive disorder, high psychological distress, and poor well-being were also observed.

Allostatic overload, which reflects the cumulative effects of stressful experiences in daily life that exceed individual coping skills (43), was found to be associated with psychological distress and poor well-being in medically ill patients; however, cluster analysis was not conducted (44). Similarly, medically ill patients with DCPR-R allostatic overload showed high rates of mental disorders and other DCPR psychosomatic syndromes (45). Using the literature as a proxy to discuss our data, demoralization (i.e., a state characterized by the patient’s awareness of having failed to meet his or her own expectations or those of others) (7) was associated with lower PWB environmental mastery, positive relations with others, purpose in life, and self-acceptance in heart-transplanted patients. However, once again, no cluster analysis was conducted (40).

The present findings showed that major depressive disorder was associated with belonging to the LC2 group. In primary care, patients diagnosed with MDD presented with at least one DCPR syndrome in 93.4% of cases—most commonly demoralization, irritable mood, and type A behavior (46). Furthermore, alexithymia, which reflects the inability to use appropriate words to describe emotions and a tendency to describe details instead of feelings (7), was found to be significantly related to depression (47) in heart-transplanted patients (40), in patients with functional gastrointestinal disorders (48), and in oncology settings (49).

Psychosomatic syndromes in the LC2 group were associated with psychological distress (i.e., mental pain, anxiety, anger-hostility, and somatization) and poor well-being (i.e., low PWB scores for environmental mastery, positive relationships with others, purpose in life, and self-acceptance; as well as low WHO-5 subjective well-being, euthymia, and PRISM measure of feeling at peace). Consistent with these findings, patients in primary care with at least one DCPR syndrome, particularly demoralization and irritable mood, showed higher levels of mental pain and lower levels of euthymia (50). Among patients with irritable bowel syndrome or irritable bowel syndrome-gastroesophageal reflux disease, those with two or more DCRP syndromes reported worse well-being and quality of life, as well as higher psychological distress and more abnormal disease behavior, compared to those with no DCPR syndromes or only one (42). Among patients with fibromyalgia, irritable bowel syndrome, migraine, coronary heart disease, or type 2 diabetes, the highest levels of anxiety and the poorest well-being were associated with having at least three DCPR syndromes (13). Similarly, among patients with type 2 diabetes, depressive symptoms were associated with at least three DCPR syndromes (13).

Although this is the first study to assess psychosomatic load in SSc, it has several limitations: (a) the study design was monocentric; (b) participation was voluntary, which may have introduced selection bias since participants could have been more motivated and in a more stable health condition than non-participants; and (c) female individuals were overrepresented due to the higher incidence of SSc in women.

However, the study also has significant strengths. It provides valuable insights into SSc from a psychosomatic point of view and enables the identification of different psychological profiles among patients with SSc. In addition, the use of the structured interview for DSM diagnosis (i.e., the MINI) and the semi-structured interview for DCPR-R (i.e., the DCPR-R-SSI) adds methodological rigor, as these tools are both standardized and diagnostic, complementing self-report assessment tools.

The DCPR is, also in patients with SSc, a powerful diagnostic tool complementary to the DSM. The number of psychosomatic syndromes occurring in the same patient may help define different and specific clinical profiles worthy of clinical attention that cannot be fully or even partially explained by biomedical or mental disorder diagnoses. A higher number of psychosomatic syndromes appears to be associated with greater psychological distress and poorer well-being. Overall, this suggests the presence of a clinical profile that can differentiate and demarcate prognostic and therapeutic differences among patients who may otherwise appear deceptively similar due to sharing the same SSc diagnosis. Such profiles are unique and specific to each patient. They should, therefore, influence clinical decision-making and treatment planning, contributing to the formulation of personalized and tailored interventions according to the biopsychosocial model (5). In addition, they may help improve treatment adherence as appropriate interventions [e.g., (51)] can have a positive impact on the psychosocial profile.

## Data Availability

The raw data supporting the conclusions of this article will be made available by the authors, without undue reservation.
